# Perspectives in Molecular Imaging Using Staging Biomarkers and Immunotherapies in Alzheimer's Disease

**DOI:** 10.1155/2013/589308

**Published:** 2013-02-05

**Authors:** Benoît Leclerc, Abedelnasser Abulrob

**Affiliations:** ^1^Department of Cellular and Molecular Medicine, Faculty of Medicine, University of Ottawa, 451 Smyth Road, Ottawa, ON, Canada K1H 8M5; ^2^Institute for Biological Sciences, National Research Council Canada, 1200 Montreal Road, Building M-54, Ottawa, ON, Canada K1A 0R6

## Abstract

Sporadic Alzheimer's disease (AD) is an emerging chronic illness characterized by a progressive pleiotropic pathophysiological mode of actions triggered during the senescence process and affecting the elderly worldwide. The complex molecular mechanisms of AD not only are supported by cholinergic, beta-amyloid, and tau theories but also have a genetic basis that accounts for the difference in symptomatology processes activation among human population which will evolve into divergent neuropathological features underlying cognitive and behaviour alterations. Distinct immune system tolerance could also influence divergent responses among AD patients treated by immunotherapy. The complexity in nature increases when taken together the genetic/immune tolerance with the patient's brain reserve and with neuropathological evolution from early till advance AD clinical stages. The most promising diagnostic strategies in today's world would consist in performing high diagnostic accuracy of combined modality imaging technologies using beta-amyloid 42 peptide-cerebrospinal fluid (CSF) positron emission tomography (PET), Pittsburgh compound B-PET, fluorodeoxyglucose-PET, total and phosphorylated tau-CSF, and volumetric magnetic resonance imaging hippocampus biomarkers for criteria evaluation and validation. Early diagnosis is the challenge task that needs to look first at plausible mechanisms of actions behind therapies, and combining them would allow for the development of efficient AD treatment in a near future.

## 1. Introduction

Late-onset Alzheimer's disease (LOAD) is an aging-associated chronic neurological disease whose etiology is not well understood. Being one of the most common forms of dementia, Alzheimer's disease (AD) is recognized as a progressive neurodegenerative disorder associated with the development of dystrophic neuritic dense-core plaques, and neurofibrillary tangles (NFTs) in the atrophic cerebral cortex of dement patients [1, 2] are hallmark neuropathological features accompanied by confusion, disorientation, memory failure, and speech disturbances towards gradual loss of mental ability progressing into reduced daily living abilities as the affected individuals aged. But whether these morphological characteristics are causative of clinical symptoms is a matter of controversy. 

Initial decline in cognition occurs more than 10 years before the first clinical AD symptoms are reported [3]. Once the illness is diagnosed, usually after 65 years of age or later, it can last from a few years up to 20 years depending on the condition severity. The prevalence of LOAD is rising proportionally with increasing world population and aging, contrasting with the less-prevalent early-onset Alzheimer's disease (EOAD) population that account for 2% of all AD cases [4]. According to the World Alzheimer Report 2009, the number of people living with AD is estimated at 36 million in 2010 and expected to increase to 66 million by 2030 and 115 million by 2050. The total cost was estimated at US$604 billion in 2010 which is about 1% of the world's gross domestic product. In the EU-15, the overall 2007 cost contributes for 68% informal care, 26% social care, 5% health care, and 1% productivity losses and, among them, UK had the highest health and social care costs associated with research funding [5]. Epidemiologic Western European studies indicate that women are more susceptible than men in developing AD between 60 and 100 years of age with an estimated odds ratio in the range of 1.6-fold higher [6]. The proportion of heredity risk factors transmitting the AD to their offspring are higher in men than women and could be explained by the fact that women are more susceptible to the influence of environmental risk and innate factors other than longevity difference that may account for a higher incidence of AD in women [7]. Gender-related disparity incidence could be associated with the decrease in estrogen hormone in postmenopausal women that may have a more effective neuroprotective role than testosterone against the development of AD where the incidence seems to be more accentuated in women compared to men in the oldest-old age range [8–10]. Increasing evidence shows that estrogen replacement therapy in combination with cholinergic-enhancing drugs could be considered as an effective therapeutic strategy for use in postmenopausal women during mild cognitive impairment (MCI) [6, 11]. 

Imaging modality technologies are increasingly employed in biomedical research. Their technology characteristics, namely, spatial resolution, temporal resolution and sensitivity of probe detection are essential to address what specific biological processes (anatomic, physiologic, metabolic, and molecular) are being conducted noninvasively [12, 13]. Nowadays scientists realized the importance of combining molecular imaging systems as one platform, for example, positron emission tomography (PET) + magnetic resonance imaging (MRI), that combine high sensitivity with spatial resolution which can be used as a more accurate diagnostic tool for evaluating molecular pathways responsible of neurologic disease progression such as AD, vascular dementia (VaD), dementia with Lewis Body (DLB), frontotemporal dementia (FTD, and Parkinson disease (PD). We anticipate that ultrasound and optical imaging are the next promising techniques that can replace high energy radioisotopes and cost effective instruments largely employed in clinical settings. As a perspective, it could be possible to combine the development of deep tissue multiphoton imaging systems with optical imaging to generate fusion image with better resolution, especially where senile plaques are produced in the cortex. Herein, the purpose of this paper is to describe current molecular imaging technologies and biomarkers employed for early clinical diagnosis as well current immunotherapies for the treatment and/or preventive approach against AD.

## 2. Cholinergic, Amyloid-**β**, Tau, and Other Hypotheses 

The major culprit responsible for the initial biological event leading to behavioural and clinical AD symptoms is the subject of intensive discussion and remains unfolded. There are currently several proposed theories that explain the underlying progressive pathogenic mechanisms scope responsible for the AD neuropathological features. When all theory models are taken together, new insight of the underlying mechanisms causing AD pathogenesis, for example, neuronal cell death and beta-amyloid (A*β*) neurotoxicity, could lead to novel treatment strategies [14]. 

The cholinergic depletion hypothesis was the first theory proposed to explain the etiology of AD pathogenesis based on the findings that memory impairment, due to loss in cholinergic transmission, could be reversed following treatment of mild-to-moderate patients with cholinergic receptor agonist (e.g., nicotinic acetylcholine receptors and muscarinic acetylcholine receptors), acetylcholinesterase inhibitors (e.g., galantamine, donepezil, and rivastigmine), acetylcholine precursors (e.g., L-alpha glycerylphosphorylcholine), and cholinergic enzymes (e.g., choline acetyltransferase) [15–19]. Functional activity studies found that acetylcholine receptors depletion compromise in basal forebrain and hippocampal neurons occur along with accumulation of A*β* oligomers, via activation of the amyloid-precursor protein (APP) processing pathway, in the hippocampus and cortex areas underpin memory and learning process [20–23]. The cholinergic deficit model in AD indicates that nerve growth factors are the principal neurotropic agent for basal forebrain cholinergic neurons and could represent the etiology multifactorial neurodegenerative disorders. For example, an elegant study using two phenotypic lines of transgenic mice, the first where TrkA signaling is inhibited, showed that antibody-neutralizing a nerve growth factor TrkA decreases cholinergic activity and induces formation of A*β* with no apparent tau neuropathology characteristics; in contrast, the second where anti-nerve growth factor mice were crossed to p75 neurotrophin receptor^exonIII(−/−)^ mice showed that antibody blocking the nerve growth factor abrogates p75 neurotrophin receptor signaling which recovered cholinergic activity and prevented A*β* production at all ages, but enhanced tau protein hyperphosphorylation [22, 24, 25]. It is hypothesized that AD pathophysiology could be triggered by simultaneous hypocholinergic tone and A*β* accumulation in which A*β* and apolipoprotein E epsilon 4 (ApoE4) could interact with alpha7 nicotinic receptors leading repression of glycogen synthase 3*β* and downstream effects towards tau protein hyperphosphorylation [26]. Past clinical trials have reported that cholinergic mechanism based drugs, such as donepezil [27], are just better than placebo-treated controls; symptomatic improvements in cognition and global functioning are temporary and offer short-term cure for AD patients carrying the ApoE4 allele. The involvement of key enzymes in lipid membrane metabolism connecting the cholinergic and glutamatergic systems such as the phospholipase A2 could play a role in cognitive alterations and neurodegenerative process in AD. Sustaining inhibition of Ca^2+^ dependent and independent phospholipase A2 during the early stages of AD may lead to A*β* formation through downregulation of cholinergic and glutamate receptors. When A*β* is already elevated during the AD pathophysiology, it could favor upregulation of Ca^2+^-dependent phospholipase A2 and secretory phospholipase A2 involved in inflammatory cytokines and oxidative stress [28]. 

**Figure 1:**
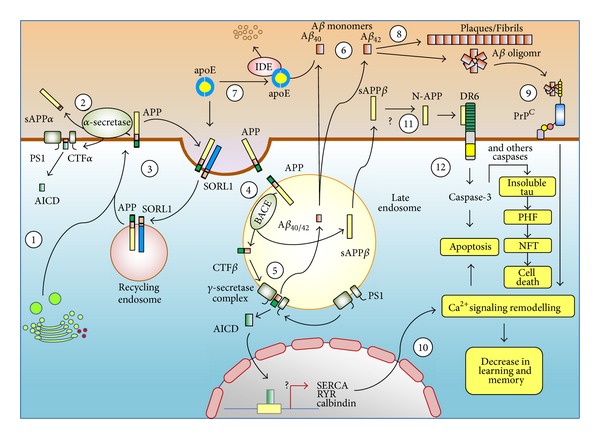
Illustration showing the implication of amyloid and tau hypotheses in the development of Alzheimer's disease. This modified illustration is reproduced with permission from [33].

Formulation of the amyloid cascade hypothesis proposed by Hardy and Higgins [29] was the most influential theory to explain why abnormal processing of A*β* constitutes the underlying mechanism responsible for the progressive development of AD pathogenesis. A modified amyloid cascade hypothesis claimed that both aging process of the brain and the associated risk factors act collectively rather than the initial A*β* deposition to exacerbate process of synapse dysfunction leading to neuronal cell death [30]. The A*β* metabolism involves a 2 steps selective cleavage of the APP by the *α*-secretase resulting in the release of the soluble short N-terminal APP*α*. The remaining C-terminal fragment-*α* is thereafter hydrolyzed by the *γ*-secretase to release the APP intracellular domain and the extracellular p3 fragment 17-40/42 [31]. The APP could be associated with sortilin-related receptor 1 (SORL1) whose complex can be internalized and enter the recycling endosomes before returning back to the surface membrane [32, 33]. These mechanisms do not lead to A*β* release. However, activating the amyloidogenic pathway leads to abnormalities in amyloid metabolism by generating short APP*β* and APP intracellular domain-*β* fragments responsible for the development of AD neuropathology. The APP cleavage is initiated by *β*-secretase to release the N-terminal short APP*β* leaving the C-terminal fragment-*β* in the membrane which is sequentially hydrolyzed by the *γ*-secretase in association with presenilin 1 and presenilin 2 enzymes complex. The *γ*-secretase cleaves the C-terminal fragment-*β* at two sites generating A*β*40 and A*β*42 which are released into the vesicle while the APP intracellular domain is released into the cytoplasm. Amyloid forms are released outside the neurons via constitutive secretory pathway whereas the C-terminal fragment-*β* will target the nucleus involving the signaling transcriptional activation [32, 33]. During the onset of AD, the transfer of autophagic vesicles to the lysosomes is impeded, thus promoting A*β* accumulation, and secondly microglia cells cannot destroy efficiently abundant A*β* monomers through phagocytosis [33]. In homozygotes, ApoE4 alleles increase the susceptibility or lifetime risk of developing AD by favoring the conversion of APP into A*β*40 and A*β*42, thus reducing their clearance via the degradation pathway [33]. Remodelling of the Ca^2+^ signaling system [34] inducing learning and memory decline involves binding extracellular A*β*42 oligomers to the cellular prion protein and alteration of ryanodine receptor expression in the nucleus following APP intracellular domain translocation. Then later, induction of apoptosis by caspase 3 could possibly involve the orphan receptor DR6 activation following its binding to the extracellular N-terminal APP (Figure 1). 

Numerous neuropathological and genetic observations demonstrate that tau hypothesis consists of abnormally elevated hyperphosphorylated form (either at threonine or serine residues) of microtubule-associated tau protein which favor NFT accumulation [35–39]. Clinical correlations have shown that tau pathology is considered as a downstream pathological event, identified as an intermediate of prerequisite A*β*-induced neurotoxic effects, preceding widespread regional neurodegeneration during the AD evolution [40–43]. The mechanism of tau phosphorylation is regulated via an imbalance from kinases and phosphates. As tau becomes hyperphosphorylated, it sequesters normal tau and other proteins causing dismantlement of microtubules involved in axonal transport. Similar to A*β*, hyperphosphorylated tau proteins become prone to self-aggregates into paired helical filaments which turn into tangles formation subsequently compromising synaptic function [32, 44]. Besides the role caspase 3 has in APP cleavage for A*β* production, it is possible a set of different caspases could contribute in tau-mediated cleavage to promote its aggregation and paired helical filaments, thus linking A*β* to neurofibrillary tangles formation during the process of AD pathogenesis [45, 46] (Figure 1). 

Following the failure of clinical trials to reduce AD progression, other factors have been proposed to be involved in this process. Among them is the emerging role of herpes simplex virus type 1 present in 90% adult brain population following childhood infection, characterized by latency and periodic reactivation causing damage over time in which apoE4 alleles carriers confer a higher risk of developing AD during aging. It has been shown that apoE4 and herpes simplex virus 1 compete with the same neuronal cell membrane receptor, called the HSPG. ApoE4 carriers, intimately binding to A*β*, influence the herpes simplex virus 1 inflammation via cytokines and iNOS and oxidative damage via lipid peroxidation processes, and as a consequence will activate the amyloidogenic pathway involving the activity of *β*-secretase and *γ*-secretase to produce more neurotoxic A*β* forms [47–49]. The homeostatic myelin repair processes represent another hypothesis underlying axonal transport interruption, axonal swellings, and neuritic plaques development, and protein deposits in A*β* and tau derivative products [50]. Some reports have recently shown that deficiency of norepinephrine in locus ceruleus projection areas is correlated with the suppression of A*β*-induced cytokine and chemokine syntheses, modulation of complement factor and impairment of microglial migration and phagocytosis, thereby reducing A*β* uptake and clearance by microglia [51, 52]. Recently, fibrinogen has been shown to interact with A*β* leading to abnormal fibrin clot formation in AD [53, 54] and could tentatively represent another hypothesis. 

## 3. Alzheimer's Disease Risk Factors and Neuropathological Aspects 

Genetic studies demonstrated that EOAD occurs with extremely high incidence in patients suffering from Down syndrome. Mutations that occur in the presenilin 1 (14q24.3), presenilin 2 (1q31-q42), and A*β* A4 precursor protein (APP; 21q21.3) [55] are responsible for autosomal-dominant early-onset familial AD. They modulate *β*- and *γ*-secretases activity that process APP cleavage for generating prematurely an age onset for the production of soluble 40 or 42 amino acid A*β* peptide. Considered as a genetic risk factor, sortilin-related receptor 1 (SORL1; 11q23.3), a low density lipoprotein receptor class A repeats, is known to regulate trafficking and processing of the APP enhancing A*β* accumulation in both EOAD and LOAD [56–58]. Although not sufficient to cause the disease by itself, inheritance of apolipoprotein E epsilon 4 (ApoE4; 19q13.2) allele constitutes a major genetic risk factor for developing EOAD as well as LOAD predisposition and hypercholesterolaemia [59, 60] and could act in synergy with other susceptible genes, for example, programmed cell death protein 4 (PDCD4) and evolutionarily conserved signaling intermediate in Toll pathway (ECSIT), in a complex interaction with environmental factors [61–65] (Table 1). The ApoE4 could be involved in cholesterol transport hindrance, diminished neuronal repair, A*β* deposition, fibrillisation, and plaque formation by acting as an A*β* interacting pathological chaperone [66, 67]. Besides APoE4, nine other candidate genes, for example, ATP-binding cassette subfamily A member 7 (ABCA7), Myc box-dependent-interacting protein 1 (BIN1), CD2-associated protein (CD2AP), CD33, clusterin (CLU), complement receptor type 1 (CR1), ephrin type-A receptor 1 (EPHA1), membrane-spanning 4A (MS4A4E/MS4A6A), and phosphatidylinositol binding clathrin assembly protein (PICALM), have been identified as AD risk loci from a three-staged meta-analysis method based on establishing the differential frequency observed between AD patients and control non-dement groups [68, 69]. Expression of ApoE4 gene with small-nucleotide polymorphism could synergistically interact with at least PICALM and, thus providing insight into their own mechanism of regulation, and serve as a diagnostic tool to predict the development of AD in nondement subjects [70, 71]. Furthermore, significant interactions of ApoE4 with SORL1 single nucleotide polymorphisms on A*β*42 cerebrospinal fluid (CSF) indicated the role that SORL1 genetic variants can have in regulating the amyloidogenic pathway [72]. Other methods have examined the relationship between small-nucleotide polymorphism loci in protein phosphatase B and calcium homeostasis modulator 1 (CALHM1), respectively, with AD quantitative biomarkers such as p-tau and A*β*42 CSF or genome-wide association study with MRI brain structure degeneration localized in temporal, parietal, and hippocampal regions [73–77]. 

Histopathological aromatic dyes staining using Thioflavin S but especially Congo red is the gold standard for diagnosing amyloid plaques because it only binds aggregated *β*-sheets [78, 79], and postmortem clinical diagnosis is still regarded as the gold standard for definitive diagnostic of AD. Other histopathological silver staining such as Bielschowsky, Bodian, or Gallyas and dyes staining such as Hematoxylin and eosin, cresyl violet, and luxol-fast blue as well as antibody specific to A*β*, phosphorylated tau, alpha-synuclein, ubiquitin, and TAR DNA-binding protein 43 (TDP-43) are routinely employed during postmortem examinations [79, 80]. Following diagnosis, neuropathological classification is assessed against A*β*, NFT, and neuritic plaque methodologies [81–83] to obtain a combined score, those Hyman et al. [84] described. The classification fall into four levels of AD neuropathological change based on the modified H. Braak and E. Braak [85] morphological staging: absence of NFT (0), low: NFT mainly in entorhinal cortex and vicinity areas (I/II), intermediate: NFT abundant in hippocampus and amygdale with some extension into the cortex (III/IV), and severe: NFT widely distributed across the neocortex (V/VI). Gradual hierarchal accumulation and distribution of NFT, neuropil threads, and dystrophic neurites take place in different brain regions during progressive AD. Anterograde A*β* expansion in brain regions has been described into 5 phases exhibiting progressive AD-related A*β* pathology [81]. In the early stages of demented elderly people, A*β* deposits are found exclusively in the frontal, parietal, temporal and occipital cortex (phase 1) which project into the hippocampal, insular, cingulate, and entorhinal cortex in addition to the amygdale where A*β* deposits occur (phase 2). Then, A*β* continues to deposit in the hypothalamus, thalamus, basal ganglia, and basal forebrain nuclei (phase 3) which are connected by multiplex afferent input regions affected from phases 1 and 2. As the disease further progress, newly senile A*β* plaques appears in the midbrain and medulla oblongata (phase 4). In the last stage (phase 5), A*β* plaques spread into the pons and cerebellum [86]. All newly affected areas receive afferent input from regions showing previously A*β* accumulation as described by Thal et al. [81].

Neuritic plaques, composed of A*β* deposits at the center of dystrophic neurites clusters containing phosphorylated tau immunoreactivity, represent another set of AD neuropathological criterion. Neuritic plaques, characterized by synapse loss and microglial activation, and NFTs are both correlated with clinical symptoms of AD. A modified method from neuritic plaque scoring system standardized protocol from postmortem assessment of dementia and normal subjects was proposed by Mirra et al. [83] is based on ranking the density of neuritic plaques in several neocortex as follows: no neuritic plaque (0), sparse (1), moderate (2), and frequent (3). Taken together, Braak NFT staging evaluation, Thal phases of A*β* deposits in neuroanatomical distribution and neuritic plaques density scoring in brain are methods used to correlate histopathological lesions during AD neuropathology changes with clinical symptoms. Molecular imaging assessment of amyloid burden in neocortex regions is a fourth method that complements neuropathological observations. A fifth method can even be utilized for discriminating soluble to aggregated peptides through biochemical assays. 

## 4. Staging Early, Middle, and Advance AD Pathogenesis Events

**Figure 2:**
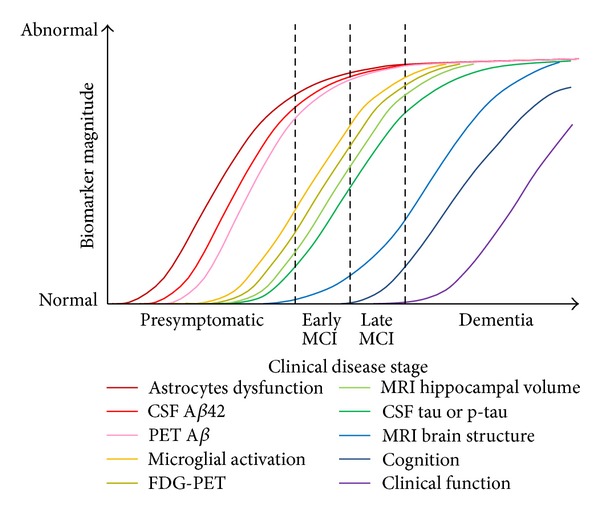
Chronobiological biomarkers to Alzheimer's disease clinical stage. This illustration is reproduced with permission from [91] and adapted from figures after [87, 101, 102].

**Figure 3:**
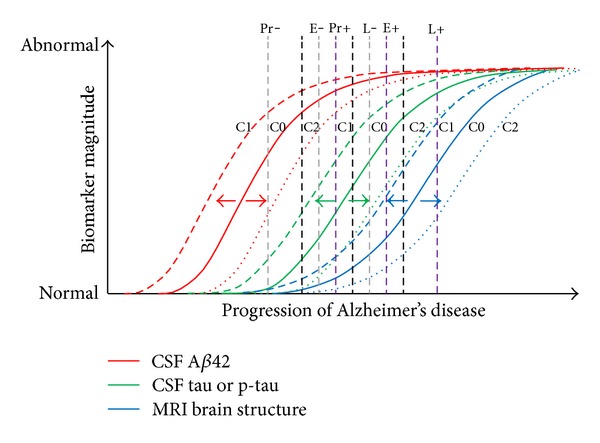
An hypothetical model showing the influence of genes, other diseases, and cognitive capacity on normal aging brain structure: C0 = CSF A*β*42, Tau or p-tau with brain structure in individuals typically observed without genetic and innate factors; C1 = CSF A*β*42, Tau or p-tau with brain structure in individuals typically observed with genetic risk loci (e.g., ApoE4) and/or together with comorbidity (e.g., cerebrovascular disease, cortical Lewy bodies, and frontal damage); C2 = CSF A*β*42, Tau, or p-tau with brain structure in individuals typically observed with high cognitive reserve or protective genetic loci (e.g., ApoE2); Pr−, Pr+, E−, E+, L−, and L+ = shifts early and late during preclinical, early, and late mild cognitive impairment stages. This modify illustration is adapted from figures in [91, 103–105].

Redefining the AD cascade hypothesis from a cholinergic, A*β* and tau point of view should be integrated into one refine model used to dissect out chronological distinct segments in pathogenic events leading to the development of stage-specific therapies. The presymptomatic period or prodromal stage refers to a pathophysiological process that is progressing towards developing cognitively and behavioural impairment of AD. The extent to which biomarkers in this period can predict a cognitively normal person who will subsequently develop clinical course of AD symptomatology remains to be clarified in the light of why some individuals never manifest the illness outcomes in their lifetime [87]. Therefore, it is critical to define the best factors contributing to the emergence of clinical impairment, so individual will benefit from early biomarker profile intervention [87]. Before the fifth decade of age, it is recognized that A*β* is low (about 5% of positive plaques number) among people who will develop LOAD. During the midfifties, the pathological cascade starts with A*β* accumulating in cognitively normal people. However, it is postulated that A*β* temporal lag between plaques depositions and clinical syndrome is estimated to be at least a decade interval [88–90]; therefore the earliest symptoms at midsixties of age represent the critical age onset of AD. It seems the temporal lag phase parallelism in A*β* deposits associated with clinical syndrome ends between 65- and 75-year old individuals. However, this does not mean all LOAD cases will have the same temporal lag as a shift in the temporal A*β* profile is observed among 65-year old individual between those who are ApoE epsilon4 noncarrier occurring in log phase versus those ApoE epsilon4 carrier who plateau [91]. There is little indication that shows how brain A*β* activation differs between these two groups over the lifespan [92]. A body of evidence highlight cognitive or brain reserve, genetic susceptibility (Figure 3) and/or environmental factors all could contribute to some extent as lifetime risk conditions of developing AD [93–98]. Nevertheless, the temporal A*β* profile is expected to be similar among the same group of individuals' going through the same neuropathological changes. In the hypothetical model of dynamic biomarkers set as a measurement of clinical dementia rating of the AD pathological cascade, the A*β*42-CSF PET is one of the most sensitive biomarker for earliest clinically detectable evidence for brain pathological changes (Figure 2). Measurement of A*β*42-CSF PET has been shown to be inversely correlated to increasing aggregation and plaque load in specific brain regions. During the lag phase when the individual is considered being in the so-called “normal status,” oligomeric soluble A*β*42 forms are quasi-absent at the beginning of life to very low level until the fifth decade of life. During the sixth or seventh decade, it is suggested that oligomeric soluble A*β*42-CSF PET increase logarithmically in cognitively normal individual corresponding to the presymptomatic phase. When oligomeric soluble A*β*42-CSF PET is already abnormally low during the stationary phase, then the individual has already MCI. However, fluorodeoxyglucose (FDG)-PET, tau-CSF and volumetric MRI may be more pertinent biomarkers at distinguishing early till late MCI. As abnormality in cognitive behaviour increases between early and late MCI and further progress during advance AD stage, volumetric MRI testing brain structure remains at present the sole biomarker measuring differentially those changes [99, 100]. 

## 5. Diagnostic PET and MRI Technologies Using AD Biomarkers and Criteria 

Molecular imaging assessment of current clinical diagnostic criteria of MCI offered good sensitivity and specificity of AD biomarkers; however it possesses deficiencies in diagnosing early-clinical stage as gold standard postmortem analysis can detect early neuropathological observations [106, 107]. To some extent, data interpretations are regarded as the degree on which cognitive function is impaired and the effect of other causes (e.g., ApoE4) could impact as lifetime risk factors on the illness progression. To complement neuropathological observations, development of molecular imaging technologies should use reference data set as a benchmark as well as neurochemical biomarkers to detect the earliest AD pathogenic events, improve classification, track progression, and assess in prediction [108, 109]. This should facilitate the diagnosis and furthermore, supported by genetic analysis of cerebrovascular risk factors and other age-related brain disease, offer comprehensive information regarding which subtype neurodegenerative disease can potentially affect each person. Standardization in diagnostic criteria is very important because it address bias-adjusted estimates of the sensitivity and specificity [110] in which molecular imaging biomarkers are applied for better defining measurements of clinical AD stages. As well, combining molecular imaging, for example, adding magnetic resonance spectroscopy to MRI measures, could result in significantly better AD classification by improving higher sensitivity and specificity of measurements translated into better biomarkers to support the clinical diagnostic of the different AD stages [111, 112]. Standardization and/or validation in diagnostic criterion and clinical AD stages have been documented for PET and MRI biomarkers [113–119]. Diagnostic AD biomarkers are important to select population for specific selection criteria (e.g., onsets of cognitive impairment evaluated by neuropsychological testing such as the mini-mental state examination), age grouping population and increasing the statistical power of clinical trials, whereas clinical trial AD biomarkers are evaluating the type of therapeutic intervention (e.g., targeting amyloid, tau, etc.), and the clinical disease stages as well as its time-course changes during progressive pathological features [120]. To help in the AD diagnostic classification, molecular imaging should look at multimodal imaging analysis as well as pathological and functional changes associated with disease stages and brain regions in order to enrich patients in clinical trials and evaluation of treatment effects [121, 122]. For example, Zhang et al. [123] showed that combining molecular imaging (volumetric MRI, Pittsburgh Compound-B (PIB)- & FDG-PET and CSF-t-tau, -p-tau and -A*β*42 biomarkers) as reported by Fjell et al.[124] would achieve a higher diagnostic accuracy when discriminating between AD (93.2%) and MCI (76.4%) with healthy patients as well as 91.5% MCI converters and 73.4% MCI nonconverters correctly classified. The single-photon emission computed tomography (SPECT) using technetium-99m labeled hexamethylpropyleneamine oxime (99mTc-HMPAO) shows superior detection to dynamic susceptibility contrast MRI at distinguishing neuroanatomical regions between normal and AD patients and can be used together as a diagnostic tool to the medial temporal lobe of MCI patients for predicting memory dysfunction associated with AD progression [125, 126]. An initial report developed by Klunk et al. [127] demonstrated a significant higher [11C]-PIB tracer signal using PET imaging in frontal cortex, but also in parietal, temporal and occipital cortex and striatum where selective binding to A*β* deposits take place in these brain regions of living AD patients compared to healthy controls. They also show a PIB inverse correlation with the FDG tracer mainly in the parietal cortex, an observation reported across different clinical stages [121, 128–130]. It remains to address at which lowest concentration threshold of A*β* corresponding to which AD clinical stage does PIB-PET produce a positive signal. Nevertheless, Ikonomovic et al. [131] showed weakly A*β* labeled with the high fluorescent 6-CN-PIB in low A*β* brain load indicating that this AD pathology case may precede the PIB detectable level. Using PET, a compound called (E)-4-(2-(6-(2-(2-(2-([18F]-fluoroethoxy)ethoxy)ethoxy)pyridin-3-yl)vinyl)-N-methyl benzenamine, also named Florbetapir, has shown to possess a half-life of 110 min versus 20 min for PIB which allows the former to accumulate significantly more in the brain regions of AD and MCI patients associated with A*β* deposits binding compared to healthy controls [132, 133]. This observation will definitively be important as an *in vivo* diagnostic agent for detecting A*β* pathology during the presymptomatic stage of AD, its related hypotheses cascade and to assess the efficacy of antiamyloid therapies under clinical development [134]. Besides measuring known and new CSF biomarkers to improve the diagnostic accuracy of A*β*42 and tau [135], clinical dementia rating (CDR) scale of 0–2 indicates that CSF biomarkers are detectable at CDR 0.5, CSF biomarkers, and FDG-PET imaging at CDR 1 and all previous ones including cerebral blood flow SPECT and MRI at CDR 2 [136]. Although hypometabolism measured by FDG-PET and brain atrophy MRI show predictive patterns, their profile reveals different rates and change according to regions and disease progression [137]. For example, Villain et al. [138] assessed the relationship between hippocampal atrophy specifically related to the cingulate bundle disruption as a factor contributing to the early posterior cingulate cortex hypometabolism and peripheral connecting network regions such as the middle cingulate gyrus, thalamus, mammillary bodies, parahippocampal gyrus, and hippocampus (whole memory Papez's circuit), as well as the right tempoparietal associative cortex in AD. Shima et al. [139] cautioned that diverse atrophy patterns exist among AD subjects, but point out a significant correlation between a subset of patients affected by posterior cingulate/precuneus atrophy with greater hypometabolic manifestation. In MCI, MRI hippocampal atrophy, tempo-parietal hypo-FDG-PET, CSF A*β*42, t-tau and p-tau, and cortical amyloid deposits [11C]-PIB biomarkers were also shown to be useful for diagnostic enrichment, designed for improving clinical trials modifying drugs therapies for AD [140]. At the present time, identifying definitive surrogate markers across the wide spectrum of AD subjects using current multimodalities imaging needs further explorative evidence. 

## 6. Methods and Approaches for the Development of Amyloid Targeted Contrast Imaging Agents 

Because A*β* can be used as a gauge for measuring early AD neuropathology, developing appropriate contrast imaging agents for *in vivo* early detection and quantitative brain deposits can benefit antiamyloid therapies. Florbetapir F18-PET has been employed to image cortical A*β* between patients with mild-to-moderate AD as well as MCI to healthy controls [133, 141]. Amyloid PET ligand florbetapir F18 has been shown to correlate closely with the localization and density of A*β* plaques identified by silver and thioflavin S staining, and immunohistochemistry [142, 143], and their results support the notion that future studies are necessary for establishing florbetapir-PET imaging as a clinical diagnosis of AD and as a reference biomarker used for the prediction of the illness progression. Similarly, Wolk et al. [144] demonstrate a concordance between flutemetamol F18-PET imaging tested in seven patients with previous biopsy, obtained from same patients at the site of ventriculo-eritoneal, used for measuring A*β* load by immunohistochemistry and histology. Yang et al. [145] were the first to report the feasibility of combining immunohistochemistry, relaxation time T2 values in regions of interest corresponding to the cortex and hippocampal (cerebellum was set as a control), and voxel-based morphometry using statistical parametric mapping to detect amyloid plaque load in AD transgenic mice following femoral injection of ultra small superparamagnetic iron oxide contrast agent (USPIO)-A*β*42. They suggested that both are quantitative methods that demonstrate prior safe use of USPIO and could be used to distinguish patients having AD from healthy controls using MRI. The question remains if this technique is capable of detecting A*β* in MCI and if can be used for prediction of AD. Moreover, MRI can look at another approach which consists at looking at the low AD signature of regional cortical thinning identified in MCI patients associated with higher risk for developing future cognitive decline and abnormally low A*β* in CSF can be useful for predicting AD [146]. No publication is currently available regarding the utility of optical imaging platform in clinical diagnostics, but detecting A*β* plaques of AD and or MCI patients noninvasively using specific contrast agents in optical technique would certainly benefit quantitative studies for amyloid neuropathological prediction. A cursory report shows that following the cerebral injection of Alexa Fluor 750-labeled antibeta amyloid mouse monoclonal antibody BAM-10, moderately superior near-infrared optical fluorescence intensity signal was observed in transgenic mice. However, Skoch et al. [147] did not test their antibody via immunohistochemistry using the same brain scanned by optical imaging. Other molecular imaging approaches have been employed in transgenic mouse. For example, polysorbate 80(PS80)-coated poly(n-butylcyano-acrylate) nanoparticles (PBCA-NP) conjugated with 6E10-Alexa488 antibody was evaluated by *in vivo* optical 2-photon imaging and gadobutrol PBCA-NP accumulation in brain parenchyma over time by MRI. Koffie et al. [148] demonstrated that PBCA-NP can be used as an efficient and safe delivery biodegradable nanocarrier of the blood-brain barrier (i.e., absorbed by the ApoE onto plasma membrane and internalized via receptor-mediated transcytosis) into the brain and to transport blood-brain barrier impermeable targeted A*β* contrast agents into the brain in sufficient amount for enhancing measurement of signal-to-noise ratio during noninvasively optical imaging and MRI scans. These prove to be clinically relevant for diagnosis A*β* cellular and neuropathological changes associated with AD. Another approach used magnetic resonance imaging-guided FUS (transcranial focused ultrasound) following intravenous tail vein administration of biotinylated BAM-10 antibody. Jordão et al. [149] notice that a low dose of 40 *μ*g BAM-10 bound to A*β* plaques in cortical regions remained associated with plaques for at least four days in brain sections and reduced plaques number density, mean size, and surface area, but did not change A*β* levels. 

## 7. Methods Development for Early Diagnosis and Therapies in AD Clinical Research 

Transgenic AD mouse model provides an excellent tool to support the amyloid cascade hypothesis as the foundation of AD pathogenesis. These animals have been designed for A*β* production, but more specifically for plaques and tangles formation [150, 151] to recapitulate the disease aspects. Although the use of transgenic mice in preclinical research is very useful, it has some limitations when assessment is conducted in human clinical trials applications as to which extent the transgenic mouse models are accurate representing sporadic human AD neuropathology when evaluating the safety and the potential use of therapeutics products [31]. The use of double transgenic 85Dbo/J mice harboring PSEN1dE9 and APPSWE transgenes devoid of tau expression is pertaining in the evaluation of cerebral amyloid hypothesis in AD mouse model. Indeed, Lewis et al. [152] observed high A*β*42 : 40 ratios among 7- to 12-month-old APP695SWE × PS1A246E animals compared to APP695SWE mice and suggest that A*β*40 represents a minor component of AD pathogenesis. Supportive of this, Qu et al. [153] designed an elegant study in which serum antibody A*β*42 trimer shows higher concentration than its monomer when associated with amyloid plaques in the brains of APP/PS1 TG mice. They suggest that A*β*42 trimer can be used as a potential therapeutic value during immunization in preventing brain A*β*42 plaque formation. 

Since the initial study of Gilman et al. [154] showing 6% of patients developed meningoencephalitis side effect due to the QS-21 added to A*β* in adjuvant vaccine, Cao et al. [155] demonstrated that adjuvant-free vaccine utilizing different A*β* carrying diverse mutations in the T-cell epitope shows a promise as effective and safe immunotherapy against AD. Other methods have shown promising efficient and safe vaccine towards inflammatory response in human and transgenic AD models [156–160]. Still more studies are needed to ascertain vaccine validation and standardization at preventing autoimmune response, vasogenic edema and microhemorrhage although detrimental side effects have been reported in transgenic mice [161–163] as well for bapineuzumab in patients [164–167]. 

**Figure 4:**
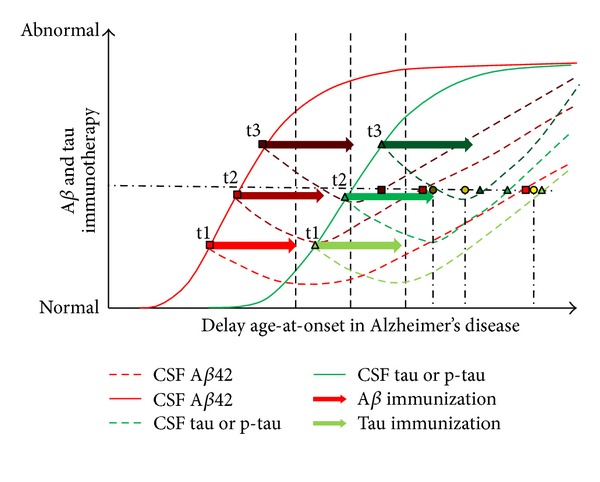
Hypothetical model illustrating the synergic effect A*β* and Tau immunotherapies combined together could have on the delay predilection of age-at-onset in progressing throughout Alzheimer's disease stages. The literature supports the notion that A*β* is not enough to treat AD symptoms, and recently tau immunotherapy may help in preventing cognitive decline in mouse AD model. This graph shows the effect each immunotherapy could have over time when giving at different pathological timelines (t1, t2, and t3). Excluding time-course, time-interval, and efficacy of A*β* and tau immunization might influence the outcome of neuropathological changes; both immunotherapies may show promise in delaying neurofibrillary tangles burden clearance into the brain, cognition decline, and clinical stages. Resulting code color glow circles are from t1 A*β* + t1 tau, t2 A*β* + t2 tau, and t3 A*β* + t3 tau, respectively.

There are currently several immunotherapy approaches that focus on effective treatments in clinical trials but should be administrated at the earliest stages to see if therapy can prevent or delay the progression of AD as suggested by Panza et al. [168] since detrimental side effects have been reported in transgenic mice [169–171]. The A*β* immunotherapy has great potential of clearing cerebral A*β* via microglia independent pathway [172], as being the most investigated molecular target for intervention in AD which is in constant development for achieving better safer and efficacy treatments. Emerging therapies should address time-course administration to observe for any differential treatment effects associated with various AD stages as described by Breitner et al. [173]. Clearance of amyloid plaques in preclinical animal model studies provides protection and reversal of neuropathology, but failed to show significant cognitive stimulation in patients with moment symptomatic changes [174–176]. Discrepancies noted between rodents and humans should be examined whether species' and strains' or races' epitope specificity and functionality occur. Among different A*β* immunotherapy approaches, the active immunization involving synthetic A*β*42 absorbed to a carrier protein and passive immunization involving monoclonal antibodies directed against A*β* synthesis elicit plaques clearance in AD subjects and more specifically for the treatment of presymptomatic to maximize potential disease modifying drugs in clinical trials [177–180]. Gamma-secretase inhibitors are known to inhibit A*β*40 in the brain, CSF, and plasma in rat [181], but significant reduction in A*β* concentrations are noticed in human plasma and CSF without clearance change [182, 183]. Another therapy called 8-hydroxy quinoline analog (PBT2) is a metal-protein attenuating compound which reduces copper- and zinc-mediated toxic oligomerization of A*β* has shown significant reduction in CSF A*β*42 concentration compared to the placebo associated with cognitive improvement during clinical trial phase 2 [184]. Crouch et al. [185] demonstrated that PBT2 having metal chaperone activity translocated extracellular zinc and copper into the cells by promoting A*β* aggregates dissolution via matrix metalloprotease 2 could provide a mechanism by which PBT2 improves cognitive function in AD subjects. Plausible mechanisms of actions propose (1) synthetic fragment of A*β* are designed at crossing the blood-brain barrier via their epitope to opsonize A*β* whereas their Fc fragment is recognized by receptor-mediated phagocytosis by microglia, (2) administrating into the vasculature system antibody targeting A*β* whose complex will be cleared from blood circulation preventing A*β* to enter into the brain to form new plaques or modified antibody that altered the blood-brain barrier permeability used to drain out A*β* from the brain into the blood circulation to enhance clearance of soluble A*β* and (3) administration of antibody inhibitors preventing amyloid accumulate in plaque [180]. Since A*β* immunotherapy shows limited clearance of tau aggregates in dystrophic neurites and neuropil threads [186, 187] and weather clearance of tau pathology modulates A*β* [188], it proposes that tau therapy should be developed separately to directly target pathological tau in AD and related tauopathies [180]. Using a mouse model of accelerated tangle development, targeting tau immunotherapy was marked with extensive abnormal tau clearance into the brain, especially the decrease of NFTs accumulation, and prevents cognitive decline in mice [188, 189]. Evidence suggested that cognitive impairment is correlated with the degree of tau pathology, and as such clearing phosphorylated tau may be a promising therapeutic approach to slow or prevent cognitive impairment in animal tauopathy model [190]. Although few clinical studies have been reported so far, active and passive immunotherapies for tau are possible at preventing the accumulated intracellulartau pathology, neurospheroids, and associated symptoms [188, 191]. Amyloid-*β* immunotherapy acts by preventing phosphorylated tau accumulation and demonstrates a link between these proteins, that both therapies should be allowed as treatment to reduce or block the progression of the illness (Figure 4). In other words, targeting early A*β* and tau pathology stages may have beneficial effects as smaller A*β* and tau assemblies should be cleared faster than mature NFTs [188]. As an exemple, apomorphine treatment in 6-month old triple transgenic AD mice shows improvement of memory function as a result of Morris water maze time-course evaluation and, in this mouse model, apomorphine has been shown to promote significantly decrease in intraneuronal A*β*, phosphorylated tau, p-53, and heme oxygenase-1 proteins in cultured SH-SY5Y cells [192]. In a near future, apomorphine may be evaluated in clinical pilot AD studies to address drug efficacy in translational medicine. 

Other therapies have shown to improve shortly cognitive performance of AD subjects. Etanercept is a fusion protein based on tumor necrosis factor alpha receptor which decreases the neuroinflammatory activity of its ligand tumor necrosis factor alpha. Clinical trial study indicates that methylthioninium chloride dissolve tau tangles during mild to moderate AD stage. Moreover, antibiotic and antiviral therapies could be used in the near future as an effective treatment. Some evidence suggests that insulin sensitizers, for example, rosiglitazone and thiazolidinedione, increase dendritic spine density of primary cortical neurons translated into cognitive recovery in a subset of AD patients [193, 194]. 

## 8. Future Prospects: Treatment and Prevention

At present the etiology that triggers progressive pleiotropic deregulated intertwined targeting pathways which contribute to synaptic lesions and thereof gradual memory loss during aging stages are based on hypothetic theory explanations, and only available treatments help on short-term to attenuate symptoms of MCI patients. Evidence point out that immunotherapy, in near future clinical trials, has the potential to slow down the progression of the disease assessed by using diagnostic PET and MRI, the two most promising molecular imaging technologies. As of December 2012, it has been found 1184 studies were conducted to treat the disease as listed in the clinicaltrial.gov website and about 20%, 25%, 15%, and 10% of these compounds are in Phase I, II, III, and IV trials, respectively. We optimistically expect that some compounds may have adequate efficacy, tolerability, and safety towards treating MCI subjects as the global economic health care burden continued to grow every day. A challenging approach, but yet feasible, would be treating patients with personalized medicine at the earliest clinical stage of dementia and follow up their cognitive evolution as compared with untreated same category patients (i.e., based on standardization of quantitative metrics and population's genetic susceptibility profile) to provide insight into the AD biomarkers' mechanisms of regulation influencing on progressive neuropathological features translated into cognitive and behaviour impairments. 

## Figures and Tables

**Table 1:** Genetics and environmental risk factors associated with human AD etiology.

Genetics^A^	
AD-causative genes	
Presenilin 1 (PSEN1)	
Presenilin 2 (PSEN2)	
A*β* A4 precursor protein (APP)	
AD-susceptible genes	
Apolipoprotein E epsilon 4 (APOE*ε*4)	
Sortilin-related receptor 1 (SORL1)	
Amyloid beta A4 precursor protein-binding, family B, member 2 (APBB2)	
Hemochromatosis (HFE)	
Nitric oxide synthase 3 (NOS3)	
PAX-interacting protein 1 (PAXIP1)	
Urokinase-type plasminogen activator (PLAU)	
Alpha-2 macroglobulin (A2M)	
Bleomycin hydrolase (BLMH)	
Myeloperoxidase (MPO)	
Angiotensin-converting enzyme (ACE)	
New AD Susceptible Loci	
Programmed cell death protein 4 (PDCD4)	
Evolutionarily conserved signaling intermediate in Toll (ECSIT)	
Phosphatidylinositol binding clathrin assembly protein (PICALM)	
Complement receptor type 1 (CR1)	
Myc box-dependent-interacting protein 1 (BIN1)	
CD2-associated protein (CD2AP)	
CD33	
Ephrin type-A receptor 1 (EPHA1)	
Clusterin (CLU)	
ATP-binding cassette subfamily A member 7 (ABCA7)	
Membrane-spanning 4A4E and 6A (MS4A6A/MS4A4E)	
Oxidized low-density lipoprotein (LDL) receptor (OLR1)	
Cholesterol 24-hydroxylase (CYP46)	

Environmental exposures^B^	

Health factors	
Food diets	
Cigarette smoking	
Alcohol consumption	
Head trauma	
Viral infections	
Systemic inflammation	
Metal and pesticide exposure	
Psychosocial factors	
Education	
Social network	
Leisure activities	
Physical activity	
Chronic stress	
Depression	
Somatic Factors	
Blood pressure	
Obesity	
Diabetes mellitus	
Cardiovascular diseases	
Cerebrovascular diseases	
Hyperlipidemia	

^A^Genetic factors associated with AD were obtained from OMIM database and [55, 61, 62, 68].

^B^Environmental factors associated with AD were obtained from [63–65].
